# Generalization of word meanings during infant sleep

**DOI:** 10.1038/ncomms7004

**Published:** 2015-01-29

**Authors:** Manuela Friedrich, Ines Wilhelm, Jan Born, Angela D. Friederici

**Affiliations:** 1 Department of Psychology, Humboldt University of Berlin, Rudower Chaussee 18, 12489 Berlin, Germany; 2 Department of Neuropsychology, Max Planck Institute for Human Cognitive and Brain Sciences, Stephanstr. 1a, 04103 Leipzig, Germany; 3 Child Development Center, University Children’s Hospital, Steinwiesstrasse 75, 8032 Zürich, Switzerland; 4 Institute of Medical Psychology and Behavioral Neurobiology and Center for Integrative Neuroscience, University of Tübingen, Otfried Müller-Str. 25, 72076 Tübingen, Germany

## Abstract

Sleep consolidates memory and promotes generalization in adults, but it is still unknown to what extent the rapidly growing infant memory benefits from sleep. Here we show that during sleep the infant brain reorganizes recent memories and creates semantic knowledge from individual episodic experiences. Infants aged between 9 and 16 months were given the opportunity to encode both objects as specific word meanings and categories as general word meanings. Event-related potentials indicate that, initially, infants acquire only the specific but not the general word meanings. About 1.5 h later, infants who napped during the retention period, but not infants who stayed awake, remember the specific word meanings and, moreover, successfully generalize words to novel category exemplars. Independently of age, the semantic generalization effect is correlated with sleep spindle activity during the nap, suggesting that sleep spindles are involved in infant sleep-dependent brain plasticity.

## Abstract (web summary)

In adults, oscillatory brain activity during sleep is related to memory consolidation. Here, the authors measure brain activity from infants who are exposed to novel word meanings, and show that infant sleep results in the retention and reorganization of recently encoded memories.

## Graphical abstract

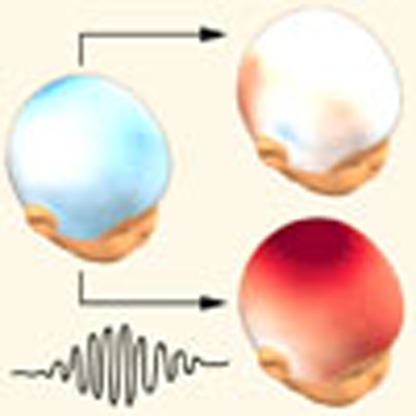

The generalization of past experience to novel instances is essential for orienting oneself in a complex environment and acting in a continuously changing world. Generalized mental categories are created online or offline, that is, either immediately during the encoding of individual experiences, or time-delayed during consolidation periods subsequent to encoding. In infancy, the generalization level of categories consistently depends on the time lag relative to encoding: immediately after exposing infants to several category exemplars, 3- to 4-month-olds finely differentiate categories at the basic level, such as dogs and cats^1^,^2^, but when testing categories established in long-term memory, even much older infants only broadly distinguish global categories, such as animals and furniture^2^. These findings indicate that infant categories undergo a process of offline generalization subsequent to their first formation. Whether this offline generalization in infancy results from passive forgetting of irrelevant details or from active reorganization of memory is still unknown and is investigated in the present study.

Sleep is the brain’s global offline state. Numerous studies in adults have evidenced that sleep promotes the consolidation of recent memories^3^,^4^. Besides the strengthening of recently encoded information, sleep-dependent memory consolidation involves the aggregation of existing memories and the creation of new knowledge. Based on an increasing body of evidence for offline generalization and gist abstraction in sleeping adults^5^,^6^,^7^,^8^,^9^,^10^,^11^, sleep is thought to actively support the reorganization of memories. In particular it has been proposed that sleep provides optimal conditions for the reactivation of recently encoded memories, the selective strengthening of overlapping information and the integration of new knowledge into the existing network of relevant neocortical memories^12^,^13^,^14^,^15^. Certain components of the sleep architecture are seen to trigger these processes in the mature brain. Slow wave activity (0.5–4 Hz) that hallmarks slow wave sleep (SWS) is thought to drive the reactivation of recently encoded hippocampus-dependent declarative memories^16^,^17^,^18^,^19^. Sleep spindles, synchronized oscillatory activity of 10–15 Hz that originate from thalamo-cortical neuronal loops, are involved in the hippocampal-neocortical dialogue during memory consolidation^20^,^21^ and can induce synaptic plasticity in the neocortex^22^. In adults, sleep spindle activity is related to memory performance^23^,^24^,^25^ and particularly associated with the offline integration of memories^26^,^27^. However, in spite of first hints for active system consolidation in infant sleep too^28^,^29^,^30^, to date, nothing is known about the role of sleep spindles and SWS in memory consolidation and offline generalization in early infancy.

The present study was intended to start filling this gap. We focused on the learning of word meanings, with the aim to characterize the infants’ generalization of word meanings as either an online or offline process, and to determine the impact of sleep and particular components of the sleep architecture on the retention and offline generalization of the newly encoded lexical-semantic knowledge. We utilized event-related potentials (ERPs) to assess the infants’ brain responses during the encoding of novel word meanings in an experimental training session and during their recognition in a memory test session about 1.5 h later. Picture–word priming effects known to index the presence of lexical-semantic knowledge in the infant ERP^31^,^32^,^33^,^34^,^35^,^36^,^37^ indicate the encoding, retention and generalization of the word meanings. To explore early developmental changes in the relation between sleep and lexical-semantic memory, we studied infants from 9 to 16 months, an age range, in which first long-term memory for word meanings is observable. Importantly, half of the infants napped in the retention period while the other half stayed awake. Group differences in the memory test ERPs of infants who napped and infants who stayed awake indeed reveal that infant memory strongly benefits from sleep. In the age range studied here, the impact of sleep on lexical-semantic memory does not change with development. The combined analyses of memory test ERPs and polysomnographic data indicate that, independent of the particular age, sleep spindles are associated with the offline generalization of word meanings. Our results suggest that, early in the immature brain, generalization results from an active reorganization of recent experiences during subsequent sleep.

## Results

### Training session

**Figure 1:**
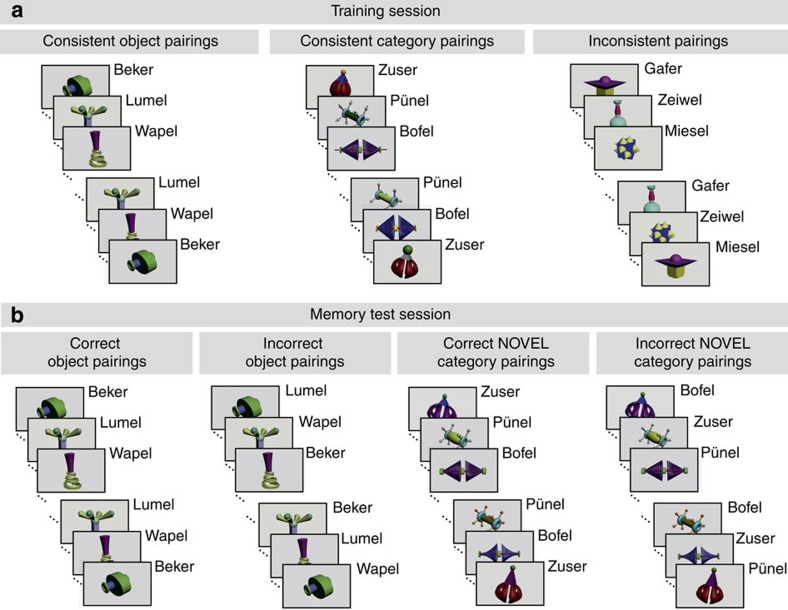
Experimental design. (**a**) In the training session, infants were presented with picture–word pairs of 80 unknown objects (16 individual objects and 64 objects forming 8 similarity-based categories) and 24 unknown words (pseudo-words). Eight distinct objects appeared in the consistent object pairing condition in which a word was paired with the same object eight times (left). The 64 category members appeared in the consistent category pairing condition in which a word was paired once with each of the 8 similar objects forming a category (middle). The remaining eight individual objects were presented eight times in the inconsistent pairing condition (control condition) in which each of the eight words was paired with each of the eight objects once (right). (**b**) In the memory test session, the stimuli of the consistent pairing conditions from the training session were presented eight times each, four times in the respective correct pairing conditions in which a pairing matched the pairing of the training session and four times in the respective incorrect pairing conditions in which a pairing violated the pairing of the training session. To test generalization in the category conditions, novel exemplars were presented.

**Figure 2:**
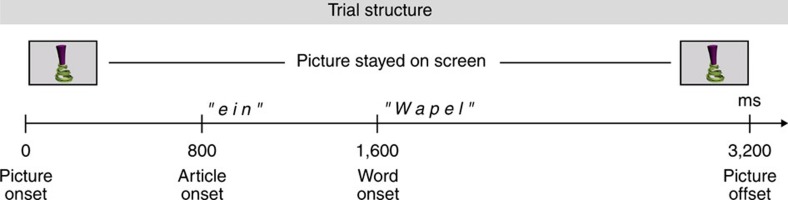
Trial structure. During the experimental sessions, infants sat on the mother’s or father’s lap in a sound-attenuated room. In each trial a coloured picture of a single object appeared on the screen for 3,200 ms. After an interval of 800 ms post picture onset, the German indefinite article *ein* (masculine/neuter) was presented to direct the children’s attention to the acoustically presented pseudo-word that followed the article presentation at 1,600 ms post-picture onset.

In the training session (Figs 1a and 2) all infants were given the opportunity to encode (a) objects as specific word meanings, (b) categories as general word meanings and as a control condition, (c) pure word forms without meanings. For this purpose they were exposed (a) to words each paired consistently with one and the same object, (b) to words paired consistently with novel, but similar objects, and (c) to words paired inconsistently with different objects.

**Figure 3:**
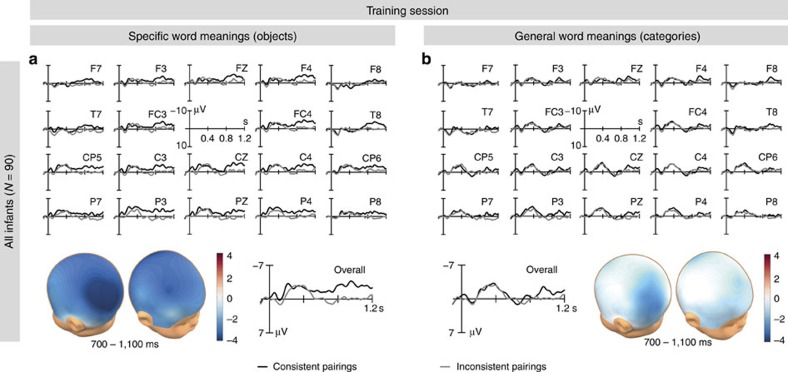
ERP data of the training session. The infant ERPs at individual electrode sites, the spatial distributions of the ERP differences between consistent and inconsistent pairings in the relevant time range and the ERP responses averaged over all sites (overall). Note that in ERP research, negativity is commonly plotted upward. (**a**) Comprehension negativity indicating the encoding of the specific word meanings (*F*_1,88_=5.361, *P*=0.023). (**b**) No evidence for the encoding of general word meanings (*P*>0.1).

Infants’ initial acquisition of specific word meanings was indicated by a late negative component (pairing: *F*_1,88_=5.361, *P*=0.023, Fig. 3a) previously reported to distinguish comprehended from unknown (or pseudo) words in the infant ERP^31^,^38^,^39^ (hence also termed ‘comprehension negativity’). As yet, this comprehension negativity has not been observed in studies with similar but less complex word learning designs. Instead, N200-500 and N400 components were seen to develop when 6- to 20-month-olds were trained with objects as specific meanings of words^34^,^35^,^37^,^40^. The different brain response here appears to reflect the much increased demand of the task (72 consistently paired objects instead of the 8 consistently paired objects of our previous studies) and might indicate a weaker or more labile state of the newly encoded specific word meanings than that built by infants exposed to less complex word learning tasks.

For words paired with category exemplars, neither the comprehension negativity (pairing: *F*_1,88_=1.177, *P*>0.1) nor any other effect indexing lexical-semantic knowledge (N200-500, N400) was evident in the ERP (Fig. 3b). The absence of a comprehension effect for categories reveals that infants did not acquire general word meanings online during the training session. However, this outcome does not imply that infants did not encode any knowledge in this condition. Similar to the object condition, they may have acquired individual object–word combinations, but this rather episodic knowledge could not cause a comprehension effect, because individual category members were not repeated.

### Memory test session

**Figure 4:**
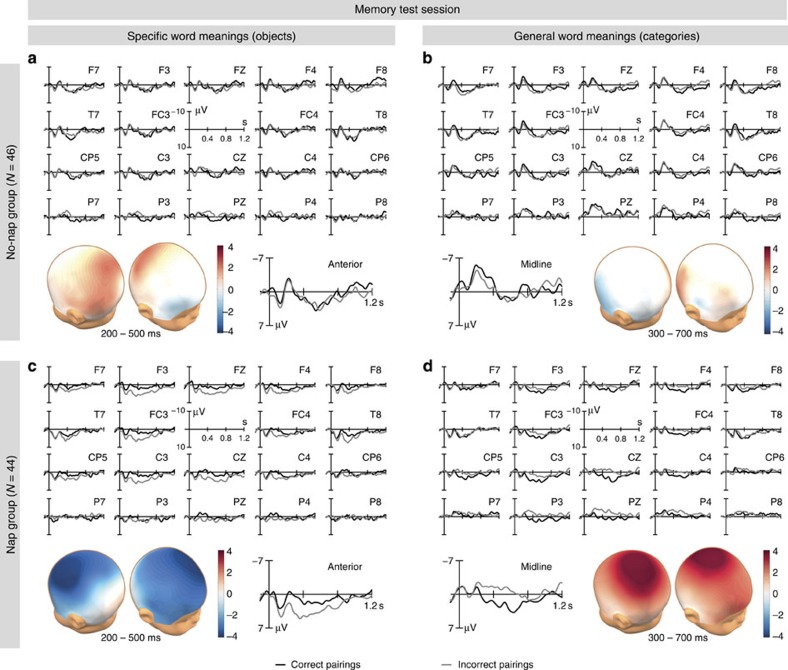
ERP data of the memory test session. The infant ERPs at individual electrode sites, the spatial distributions of the ERP differences between correct and incorrect pairings in the relevant time ranges, and the ERP responses averaged over anterior sites (anterior) or over midline sites (midline). (**a**) No evidence for the retention of specific word meanings in the no-nap group (*P*>0.1). (**b**) No evidence for the creation of general word meanings in the no-nap group (*P*>0.1). (**c**) N200-500 word form priming effect indicating long-term memory for the object–word associations in the nap group (anterior: *T*_43_=2.421, *P*=0.020; central: *T*_43_=2.259, *P*=0.029). (**d**) N400 semantic priming effect indicating the existence of newly created general word meanings in the long-term memory of the nap group (midline: *T*_43_=−2.540, *P*=0.015). Note that the N400 is commonly calculated as difference between unprimed and primed conditions, but to visualize the different polarity of the infant priming effects (that is, N200-500 increase and N400 decrease) we uniformly used the difference between correct and incorrect pairings for the illustration of the spatial distributions.

In the memory test session (Fig. 1b) infants were exposed to the same (correct) and different (incorrect) pairings as in the training session. As expected, the brain responses of infants who had stayed awake during the retention period and infants who had napped subsequently did not differ in the training session (*P*>0.1 for all interactions involving pairing and group), but they were distinct in the memory test session about 1.5 h later. The different memory test ERPs of the nap and no-nap groups (Fig. 4) revealed that the brain state during the retention period affected both the retention of the specific word meanings (200–500 ms: pairing × group: *F*_1,87_=4.033, *P*=0.048) and the generalization of more abstract word meanings (300–700 ms: pairing × hemisphere × group: *F*_2,174_=4.042, *P*=0.022). The relevant effects were not modulated by the infants’ age (*P*>0.1 for all interactions involving pairing and age).

The ERPs of infants who did not nap neither indicated memory for specific word meanings (pairing: *F*_1,45_=0.596, *P*>0.1, Fig. 4a) nor showed any sign of generalizing words to novel category exemplars (pairing: *F*_1,45_=0.005, *P*>0.1; pairing × hemisphere: *F*_2,90_=1.478, *P*>0.1, Fig. 4b). These infants had forgotten the specific word meanings acquired in the training session and had not created general word meanings offline within the retention period.

In contrast, the two brain responses known to indicate the presence of lexical-semantic knowledge in the infant ERP, namely the N200-500 and the N400 components^31^,^32^,^33^,^34^,^35^,^36^,^37^,^41^, reveal that infants who had napped in the retention period had transferred the newly encoded word meanings from temporary to long-term memory. The N200-500 thought to reflect improvements in word processing by word form priming^31^,^32^,^33^,^36^,^40^ was increased for words that had been correctly paired with individual objects when compared with words incorrectly paired with objects (pairing: *F*_1,43_=4.726, *P*=0.035; pairing × region: *F*_2,86_=2.751, *P*=0.091; anterior: *T*_43_=2.421, *P*=0.020; central: *T*_43_=2.259, *P*=0.029; posterior: *P*>0.1, Fig. 4c). This N200-500 priming effect indicates long-term memory for the recently acquired specific word meanings. Note that the N200-500 does not evidence higher-level semantic representations, since it may also be triggered by more direct associations between the visual representations of individual objects and the auditory representations of words. The presence of N200-500 word form priming without a co-occurring N400 semantic priming effect indeed suggests that the specific word meanings encoded in the object condition were retained as pure object–word connections.

On the other hand, the N400 well-known to evidence the involvement of higher-level semantic processing stages in both the mature^42^,^43^ and the immature^31^,^32^,^33^,^34^,^35^,^36^,^37^,^41^ brain was decreased for words that had been correctly paired with novel category exemplars (pairing: *F*_1,43_=4.169, *P*=0.047; pairing × hemisphere: *F*_2,86_=4.137, *P*=0.024; pairing midline: *T*_43_=−2.540, *P*=0.015, Fig. 4d). As is common, the N400 priming effect had a maximum over the central and parietal-midline regions (pairing: *T*_43_=−2.680, *P*=0.010 (CZ), *T*_43_=−2.315, *P*=0.025 (PZ)). This clearly semantic effect revealed that the sleeping infant brain had integrated recent experiences with individual category members into new semantic memory structures that represent the general meanings of words.

### Polysomnographic results and correlation analyses

On average, infants of the nap group slept for 45 min (s.d. 21 min). During their nap, infants spent most time in non-rapid eye movement (NonREM) sleep with more than 75% (mean 76.31%, s.d. 12.76%) in SWS (stages 3 and 4), and only about 5% (mean 5.29%, s.d. 7.81%) in REM sleep (Table 1).

**Figure 5:**
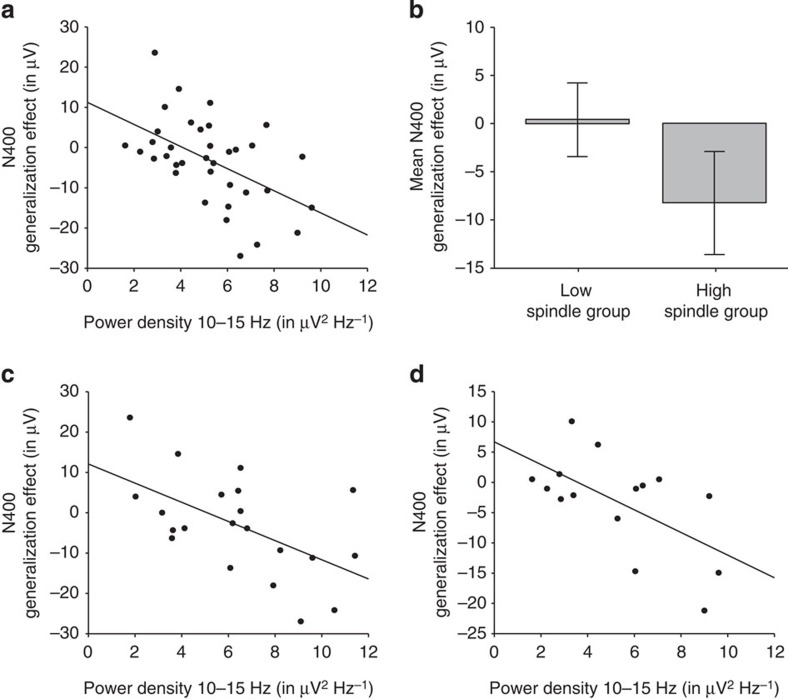
The relation between sleep spindles and generalization. (**a**) Correlation between parietal EEG power (mean over P3 and P4) in the spindle frequency range (10–15 Hz) during NonREM sleep in the retention period and the parietal N400 generalization effect (the ERP difference between incorrect and correct pairings at PZ) during the memory test session (*r*=−0.51, *P*=0.001; note that due to the negative polarity of the N400 effect the correlation is also negative). (**b**) N400 generalization effect (mean of CZ and PZ) in the group with high power in the spindle frequency range (high spindle group: *T*_17_=−3.089, *P*=0.007) and in the group with low power in the spindle frequency range (low spindle group: *P*>0.1) with error bars (±2 s.e.m.). Spindle groups did not differ in age (*T*_34_=0.432, *P*>0.1). (**c**) Correlation between EEG power in the spindle frequency range (10–15 Hz) and the N400 generalization effect for the younger group (*N*=21, mean age 10 months 22 days, s.d. 15 days, *r*=−0.56, *P*=0.008). (**d**) Correlation between EEG power in the spindle frequency range (10–15 Hz) and the N400 generalization effect for the older group (*N*=15, mean age 15 months 3 days, s.d. 23 days, *r*=−0.60, *P*=0.018).

To determine the impact of sleep spindles and slow wave activity on the retention and offline generalization of the new word meanings, we conducted specific correlation analyses between the sleep parameters of interest and the ERP effects during the memory test session. Electroencephalography (EEG) power in the spindle frequency range (10–15 Hz) was significantly correlated with the parietal N400 priming effect that indexes the presence of general word meanings, with this effect being most pronounced for spindle activity over the parietal region (*r*=−0.51, *P*=0.001 for spindle activity parietal; *r*=−0.44, *P*=0.006 for spindle activity central and *r*=−0.39, *P*=0.018 for spindle activity frontal; Fig. 5a). When splitting the nap group into low spindle group and high spindle group (median split due to power density), the N400 generalization effect was verifiable only in the high spindle group (pairing × spindle group: *F*_1,34_=7.896, *P*=0.008; pairing × region × spindle group: *F*_1,68_=3.151, *P*=0.061; high spindle group posterior: *T*_17_=−3.621, *P*=0.002; central: *T*_17_=−2.597, *P*=0.019, anterior: *P*>0.1; low spindle group: *P*>0.1, Fig. 5b). Analyses of discrete spindles revealed that the N400 generalization effect was not correlated with spindle density, spindle count or spindle length (*P*>0.1), but was correlated with the mean spindle amplitude (*r*=−0.42, *P*=0.009).

To uncover possible age effects, we divided the whole nap group into two age groups. Age subgroups differed neither in total sleep duration and the proportion of individual sleep stages nor in any of the spindle measures (*P*>0.1). Separate correlation analyses of the age subgroups (younger group from 9 to 12 months: *N*=21, mean age 10 months, 22 days, s.d. 15 days; older group from 13 to 16 months: *N*=15, mean age 15 months, 3 days, s.d. 23 days) revealed that the correlation between sleep spindle activity and semantic generalization is present within both age ranges (younger group: *r*=−0.56, *P*=0.008; older group: *r*=−0.60, *P*=0.018, Fig. 5c,d). Moreover, a partial correlation analyses with controlling the effect of age (in days) did not substantially change the correlation within the whole group (*r*=−0.51, *P*=0.002 for spindle activity parietal; *r*=−0.45, *P*=0.007 for spindle activity central and *r*=−0.39, *P*=0.024 for spindle activity frontal), confirming that the relation between sleep spindles and semantic generalization holds independent of age.

None of the spindle measures were significantly associated with the N200-500 priming effect indexing the retention of specific word meanings. In addition, slow wave activity was not correlated with any of the ERP effects.

## Discussion

The results provide strong evidence that infant sleep accomplishes the retention and reorganization of recently encoded memories. Depending on the word learning condition, a short nap preserves specific word meanings on one hand, while abstracting general word meanings on the other hand. Thus, when infants are given the possibility to acquire specific and general knowledge in parallel, both the veridical retention of distinct individual memories and the formation of new memories generalized from similar experiences are strongly promoted by post-encoding sleep.

Unlike the present study, the only two previous studies on sleep-dependent memory consolidation in infancy tested specific and general knowledge for the same set of items. In these behavioural studies, 15-month-olds who had napped in the consolidation period were found to be sensitive to an abstract grammatical pattern, while infants who had stayed awake showed veridical memory for familiar grammatical strings^28^,^29^. Thus, when the consolidation of specific and general knowledge competes, infant sleep appears to favour general knowledge.

In older children, sleep rather favours the consolidation of specific knowledge with contextual details. In a study with 2.5-year-olds, only those children who had stayed awake during the retention period generalized words to novel category exemplars presented in novel contexts, whereas children who had napped during the retention period did not show such generalization behaviour^44^. These data together with the present findings suggest developmental changes in the preferred sleep-dependent memory consolidation across early childhood. However, the involvement of context changes (background colour and texture) in this prior study may be critical for the outcome in the older children, since different stages of language development require different sensitivity to contextual changes. While context is largely irrelevant for the learning of object labels, context becomes much more relevant and should not be ignored when children acquire utterances describing relations between contextually bound elements, such as verbs, phrases and sentences. Thus, the different finding in toddlers might not reflect a developmental shift in sleep-dependent memory consolidation per se, but rather the adjustment of sleep-dependent memory consolidation to the developmentally relevant characteristics of the environment.

Here we provide first evidence for the generalization of word meanings during infant sleep. The basis for such sleep-dependent generalization has been suggested to result from the replay of overlapping memory representations and the selective strengthening of shared elements^12^,^13^,^14^,^15^. In current consolidation models, the slow oscillations during SWS play a major role in the reactivation of recent declarative memory content^16^,^17^,^18^,^19^. In the infants of the present study, we did not find correlations between slow wave activity during sleep and ERP effects during the memory test. This may indicate functional differences of SWS across development, as also suggested by differences between infants and adults in the distributions of slow wave activity across sleep stages^45^ and by changes in the topography of slow wave activity during development^46^. Alternatively, the missing association between slow wave activity and memory consolidation in our study might simply reflect a ceiling effect as all infants spent most of their nap time in SWS.

Sleep spindles are considered to enhance plasticity in the neocortex by inducing synaptic short- and long-term potentation^22^. In our infant sleep group, the power in the spindle band during NonREM sleep was correlated with the N400 semantic generalization effect in the memory test session, suggesting that infant sleep spindle activity had been involved in the offline generalization of new word meanings. This finding is in line with studies reporting relations between spindle activity and sleep-dependent memory improvements in adults^23^,^24^,^25^. The particular result that infant sleep spindles are correlated with the integration of specific into generalized word meanings, but not with the veridical retention of word meanings parallels findings in adults, according to which sleep spindles were associated with the integration of newly learned word forms into the mental lexicon, but not with the pure retention of the newly learned words^26^,^27^. Our data provide first evidence that the supposed crucial role of sleep spindles for aggregating recent memories and for anchoring new knowledge into neocortical memory structures also holds for infancy. The here observed first correlation between sleep spindles and knowledge generalization in infants from 9 to 16 months of age suggests that the spindle-driven neural mechanisms that realize sleep-dependent brain plasticity are effectively established early in life in the immature brain. The relation between infant sleep spindles and semantic generalization moreover speaks for the notion that offline generalization during infant sleep results from an active reorganization of memory and not from passive forgetting of irrelevant features.

Here we show that, like in adults and older children^47^,^48^,^49^,^50^, a short nap is sufficient for infant memory consolidation to occur. During their nap, infants spent on average 42 min in NonREM sleep. This corresponds to the mean duration of a NonREM period during infants’ overnight sleep, which has been found to vary from 37.5 min in 9-month-olds to 47.2 min in 16-month-olds^45^,^51^. Thus, our results provide clear evidence that a single NonREM period substantially promotes declarative memory in infancy.

In the present study, we cannot entirely rule out that groups differed with respect to variables that can affect memory performance in a word learning task, for example, status of brain maturation and cognitive development. However, lacking group differences for early life variables associated with later development (that is, gestational age at birth, birth weight and Apgar scores) together with the large group sizes make substantial differences unlikely. Importantly, in the here studied age range from 9 to 16 months, not any effect was modulated by the infant’s age. However, age effects should have been observed if individual variations in brain maturation and cognitive development had an impact on the relation between sleep and memory within the covered developmental period. Moreover, the nap and no-nap group did not differ in the encoding of object–word or category–word pairings, which would have been expected if groups really differed in any variable confounding word learning. Furthermore, the two seminal studies on sleep-dependent memory consolidation in infancy provided behavioural evidence for a sleep-induced generalization in 15-month-old children^28^,^29^, which strengthens the claim that the generalization effect observed in our study was indeed caused by post-encoding sleep. And finally, the fact that the generalization effect was associated with sleep spindle activity is a strong argument to link the reported offline generalization to sleep rather than to any other variable that might have differed between the groups. The finding that the generalization effect was only observed in the group with high-amplitude spindle activity but not in the group with low-amplitude spindle activity suggests that a certain level of spindle power is necessary for the infant’s memory system to perform substantial generalization. Why some infants generate such high-amplitude spindle activity while others do not is yet to be discovered.

In summary, our results show that sleep affects memory organization very early in human life, at a time when memory grows rapidly and extensively. While the awake infant brain shortly forgets new experiences, the sleeping immature brain preserves recently learned distinct word meanings in a veridical way. At the same time, the brain’s global offline state provides mechanisms that aggregate similar word meanings and create semantic categories as novel meanings of recently experienced words. This sleep-dependent formation of new infant memories is driven by sleep spindles. The finding of semantic generalization during infant sleep, particularly during periods of high spindle activity, advance our understanding of how the developing brain reorganizes itself and creates semantic memories from individual episodic experiences.

## Methods

### Participants

Overall, 90 infants (38 females) aged from 9 to 16 months (mean age 385 days, s.d. 70 days, min 291 days, max 511 days) contributed to the analyses. Inclusion criteria to participate in the study were monolingual German speaking parents, birth weight >2,500 g, no known visual deficit and no hearing impairment. All parents gave informed consent before participation. The study was approved by the ethics committee of the Humboldt University Berlin.

Prior to the lab visit, infants were assigned to either the nap group or the no-nap group. Infants of the nap group were scheduled at a time when they were expected to take a nap within an hour. Infants of the no-nap group were scheduled at a time when they were expected not to take a nap within the next 3 h. The nap group (*N*=44, 19 females, mean age 385 days) and the no-nap group (*N*=46, 19 females, mean age 385 days) did not differ in age (*T*_88_=0.046, *P*>0.1), gestational age at birth (*T*_87_=−1.448, *P*>0.1), birth weight (*T*_88_=−0.499, *P*>0.1) and Apgar scores (available in 80% of the sample, median in both groups: 9/10/10 for the 1/5/10 min Apgar score, *P*>0.1 for all three scores).

An additional 43 infants were measured but were excluded from the analyses because of providing too few artifact-free trials in one of the experimental conditions due to excessive body movements (23), because their behaviour (sleep/wakefulness during the retention period) did not match their group assignment (10), due to lack of interest (the infant’s attention towards the monitor was rated <50%) during either the training or the memory test session (7), due to sleepiness (2) or because of very noisy ERP data (1).

### Stimuli

Visual stimuli were 112 coloured illustrations, 16 were pictures of individual pseudo-objects and 96 were pictures of pseudo-objects belonging to 8 different similarity-based categories. The 12 exemplars of a category differed in the features of their global shape (for example, in the width-to-height ratio), in the presence/absence and shape of specific details and in the colour of either the global shape or specific details (see Fig. 1).

Auditory stimuli were 24 disyllabic pseudo-words. All pseudo-words were phonotactically legal in German, were stressed on the first syllable as common in German, had a consonant–vowel onset and had typical masculine or neuter endings. They were spoken slowly by a young woman with a mean duration of 809 ms per word, digitized at a rate of 44.1 kHz, and presented through loudspeaker with an intensity of ~65 dB sound pressure level. Three lists each with eight pseudo-words were created and assigned to the experimental conditions of the training session. The assignment of the lists to the conditions was balanced between subjects.

### Experimental design

The training session had three experimental conditions presented in counterbalanced order to each infant, that is, trial types were intermixed. In the *consistent object pairing condition* (Fig. 1a left), eight individual object–word pairs were each repeated eight times, allowing the learning of specific word meanings. In the *consistent category pairing condition* (Fig. 1a middle), eight category–word pairs were also repeated eight times, each with eight individual category exemplars presented only once. This condition was designed to enable the building of category representations and the formation of general word meanings. To assess pure repetition effects in the *inconsistent pairing condition*, eight objects and eight words were presented in pairs eight times. In this control condition, each object was paired with each word once (Fig. 1a right) such that word forms but not word meanings could be learned. The training session lasted for 10.5 min.

Approximately 1–2 h were scheduled for the retention period. The mean duration of the retention period (the time between the end of the training session and the beginning of the test session) was 84.3 min (s.d. 29 min, nap group and no-nap groups did not differ: *T*_88_=−1.246, *P*>0.1). Between the training and test sessions, parents usually left the lab and took the baby for a walk in the pram. Most children of the nap group slept in their pram, and most children of the no-nap group looked around while their parents were walking with them through the park. In bad weather, infants slept in their pram in the lab, and wake infants played with toys primarily engaging perceptual or motor activities such as a marble run, soap bubbles or toy blocks.

The test session had four counterbalanced conditions, that is, the testing of specific and general word meanings was intermixed. In the *correct object pairing condition* (Fig. 1b left) the same object–word pairs as in the consistent object pairing condition of the training session were presented four times. In the *incorrect object pairing condition* (Fig. 1b middle left), the same objects and the same words were presented, but each time in a novel pairing that violated the pairing of the training session. The *correct category pairing condition* (Fig. 1b middle right) and the *incorrect category pairing condition* (Fig. 1b right) were designed in the same way, except that four novel exemplars were presented for each category. To equal the ratio of individual objects and category members in the training and memory test session, the pairs of the inconsistent condition of the training session were also presented in the test session, although not analyzed here. The test session lasted for 10.5 min.

### ERP data acquisition and processing

For ERP analyses, EEG was recorded with a PORTI-32/MREFA (Twente Medical Systems) at 21 electrode sites and digitized online at a rate of 500 Hz. Offline, EEG was re-referenced to the average of recordings from left and right mastoids, and filtered between 0.5 and 20 Hz (−3 dB cutoff frequencies of 0.62 and 19.88). Trials exceeding a s.d. of 70 μV within a gliding window of 300 ms at any electrode site were rejected.

ERPs were analyzed time locked to word presentations. To observe encoding effects, we focused on trials from the second half of the training session, that is, 5th–8th presentations of a word stimulus. To observe memory effects, all correct and incorrect trials of the memory test session (each four presentations of a word stimulus for correct and incorrect conditions) were analyzed.

For each condition, epochs of 1,200 ms from word onset were averaged, according to a 100-ms pre-stimulus baseline. A minimum of five artifact-free trials for the conditions in the second half of the training session and a minimum of seven artifact-free trials for the conditions of the memory test session were required for an individual average to be included in further analyses. On average, 14 trials contributed to an ERP condition of the training session, and 17 trials to that of the memory test session.

According to both visual inspection of the ERP and latencies of known infant ERP components, mean amplitudes in the time window from 700 to 1,100 ms were calculated to evaluate the comprehension negativity, from 200 to 500 ms for the N200-500 priming effect and from 300 to 700 ms for the N400 priming effect.

For the statistical analyses, lateral electrode sites were combined into regions-of-interests. The averaged ERPs at F7, F3 and FC3 formed the left anterior region; at F8, F4 and FC4 the right anterior region; at C3, T7 and CP5 the left central region; at C4, T8 and CP6 right central region; at P3, P7 and O1 the left posterior region and at P4, P8 and O2 the right posterior region. To examine the encoding of word meanings, three-way repeated measures analysis of variance (ANOVA) with pairing (consistent versus inconsistent), hemisphere (left, midline and right) and region (anterior, central and posterior) as within-subject factors and group (nap versus no-nap) as between-subject factor were performed for the object and category conditions of the familiarization session. To evaluate long-term memory, three-way repeated measures analysis of co-variance (ANCOVA) with pairing (correct versus incorrect), hemisphere and region as within-subject factors, group as between-subject factor and age as a covariate were performed for the object and category conditions of the memory test session. Following significant interactions with group, data of nap and no-nap groups were analyzed by separate ANOVAs. Whenever necessary, mean amplitudes of individual hemispheres, regions-of-interests or midline electrodes were calculated and tested. In all ANCOVAs and ANOVAs, Greenhouse–Geisser-adjusted *P* values are reported whenever degrees of freedom are >1.

### Sleep recordings and spindle activity

Infants’ sleep was recorded using a portable amplifier (SOMNOscreen EEG 10-20, Somnomedics, Kist, Germany). EEG recordings were obtained with electrodes attached at F3, F4, C3, C4, P3 and P4, all referenced to CZ. Electrooculographic and electromyographic recordings were bipolar. EEG signals were sampled at a frequency of 256 Hz and filtered between 0.03 and 35 Hz. Offline, recordings were visually scored according to standard criteria^52^,^53^. For each nap, total sleep time and the time as well as the percentage of total sleep time spent in the different sleep stages were determined. Sleep stages are wake, sleep stages 1, 2, 3 and 4 (SWS was calculated as the sum of sleep stage 3 and 4) and REM sleep. In two subjects, sleep stages could not be determined because of too much artifacts in the sleep EEG.

Power spectral analysis of the EEG signal was performed using Fast Fourier Transformation on all recording sites and for periods of NonREM sleep (sleep stages 2, 3 and 4). The spectra were calculated for successive 8-s (2,048 data points) artifact-free intervals using a Hanning window to taper the data. Power density (μV^2^ Hz^−1^) was computed for the spindle frequency band (10–15 Hz) and the slow wave activity band (0.6–4 Hz). Average power in these bands was calculated first over all bins in the frequency range of interest; then averages were calculated for the succeeding 8-s intervals. To analyze the relationship between spindle activity and the ERP measures of memory performance, means over the two frontal, two central and two parietal electrodes, respectively, were calculated. Eight subjects had to be excluded from this analysis because of substantial artifacts in the sleep EEG. Note that the high number of excluded subjects is mainly due to the fact that infants frequently move during sleep.

Spectrograms were also used to identify spindles in NonREM sleep at C3. Spindles were detected automatically using a custom-made software tool (SpindleToolbox V.3) that was based on an algorithm adopted from previous studies^20^. Briefly, first the power spectrum of each subject was calculated enabling the user to visually detect the peak of the sigma frequency band in each individual (mean±s.d. 13.86±0.82 Hz). Then, the root mean square (RMS) of the band-pass filtered signal in the range of±1.5 Hz around the detected spindle peak of each 200-ms interval was calculated and the events were counted for which the RMS signal exceeded a threshold of 1.5 s.d. for 0.5–3 s. No individual spindle peak could be detected in six infants and therefore these subjects were excluded from the correlation analysis. To analyze the association between spindle measures and the ERP measures of memory performance, correlation analyses were conducted using Pearson’s correlation coefficient.

## Author contributions

M.F. and I.W. designed the study; M.F. analyzed the ERP data; I.W. analyzed the sleep data; M.F. and I.W. interpreted the data and wrote the manuscript; J.B. and A.D.F. discussed the results and critically revised the manuscript.

## Additional information

**How to cite this article:** Friedrich, M. *et al.* Generalization of word meanings during infant sleep. *Nat. Commun.* 6:6004 doi: 10.1038/ncomms7004 (2015).

## Figures and Tables

**Table 1:** Sleep data.

**Total sleep time (min)**	**44.79±21.13**
Sleep stages—time in min	
Wake	1.36±1.51
Stage 1	1.13±0.90
Stage 2	5.93±6.40
Stage 3	11.45±6.80
Stage 4	23.19±12.40
REM	3.08±4.61
	
Sleep stages—% of TST	
Wake	2.83±3.59
Stage 1	3.00±2.92
Stage 2	12.09±9.75
Stage 3	25.91±13.28
Stage 4	50.39±18.15
REM	5.29±7.81
	
Spindles	
Peak frequency (in Hz)	13.86±0.82
Count (total number)	130.79±63.22
Density (average number (30sec)^−1^)	1.82±0.32
Length (in sec)	0.96±0.12
Amplitude (in μV)	32.81±10.45

REM, rapid eye movement; TST, total sleep time.

Mean (±s.d.) TST and time spent awake, in NonREM sleep stages 1, 2, 3, 4 and REM sleep in minutes and percentage of total sleep time (*N*=42), and spindle parameters (*N*=38).
